# Determining Validity and Reliability of an In-Field Performance Analysis System for Swimming

**DOI:** 10.3390/s24227186

**Published:** 2024-11-09

**Authors:** Dennis-Peter Born, Marek Polach, Craig Staunton

**Affiliations:** 1 Swiss Swimming Federation, Swiss Development Hub for Strength and Conditioning in Swimming, 3048 Worblaufen, Switzerland; 2 Swiss Federal Institute of Sport Magglingen, Department for Elite Sport, 2532 Magglingen, Switzerland; 3 Faculty of Science and Medicine, University of Fribourg, 1700 Fribourg, Switzerland; 4 Umimplavat.cz, Analysis and Consultation for Swimming Technique and Race Performance, 18000 Prague, Czech Republic; marek@umimplavat.cz; 5 Department of Social Sciences in Kinanthropology, Palacky University Olomouc, 77900 Olomouc, Czech Republic; 6 Department of Environmental and Bioscience, School of Business, Innovation and Sustainability, Halmstad University, 30118 Halmstad, Sweden; craig.staunton@hh.se

**Keywords:** competitive swimming, elite athlete, junior, youth

## Abstract

To permit the collection of quantitative data on start, turn and clean swimming performances in any swimming pool, the aims of the present study were to (1) validate a mobile in-field performance analysis system (PAS) against the Kistler starting block equipped with force plates and synchronized to a 2D camera system (KiSwim, Kistler, Winterthur, Switzerland), (2) assess the PAS’s interrater reliability and (3) provide percentiles as reference values for elite junior and adult swimmers. Members of the Swiss junior and adult national swimming teams including medalists at Olympic Games, World and European Championships volunteered for the present study (n = 47; age: 17 ± 4 [range: 13–29] years; World Aquatics Points: 747 ± 100 [range: 527–994]). All start and turn trials were video-recorded and analyzed using two methods: PAS and KiSwim. The PAS involves one fixed view camera recording overwater start footage and a sport action camera that is moved underwater along the side of the pool perpendicular to the swimming lane on a 1.55 m long monostand. From a total of 25 parameters determined with the PAS, 16 are also measurable with the KiSwim, of which 7 parameters showed satisfactory validity (*r* = 0.95–1.00, *p* < 0.001, %-difference < 1%). Interrater reliability was determined for all 25 parameters of the PAS and reliability was accepted for 21 of those start, turn and swimming parameters (ICC = 0.78–1.00). The percentiles for all valid and reliable parameters provide reference values for assessment of start, turn and swimming performance for junior and adult national team swimmers. The in-field PAS provides a mobile method to assess start, turn and clean swimming performance with high validity and reliability. The analysis template and manual included in the present article aid the practical application of the PAS in research and development projects as well as academic works.

## 1. Introduction

Performance analyses are used to disassemble complex competition performances into their individual components and key performance indicators. Assessing each component individually helps to determine a swimmer’s strengths and weaknesses, establish training strategies and monitor performance progression [1,2,3]. A major challenge is to retrieve those data without interfering with swimming technique, streamlining or fluid dynamics. For instance, previous research has shown that the artificial environment of a swimming flume significantly alters stroke mechanics, particularly during the water catch phase at the start of the arm stroke, as the water flows against the swimmer rather than the swimmer moving through still water [4,5,6]. Additionally, the absence of turns with the associated breath-holding during the underwater phase removes a particularly challenging race component [7], which has recently been determined as a distinguishing factor for swim races [1,8,9,10]. Therefore, swimming in a flume provides advantages for respiratory and metabolic measurements but is inadequate for assessment of start, turn and clean swimming performance [11].

Methods such as surface electromyography, tethered swimming or wearable technology can be used in a normal pool environment [4,12,13]. Particularly, wearable sensory technology is inexpensive, lightweight and valid, particularly for the assessment of swimming technique [14,15,16]. However, start and turn performances require further methods, especially since recent studies showed the importance of those acyclic phases for the results of modern swim races [8,17]. Additionally, comparability to data from race results, i.e., kinematic data from the overwater phases and split times, facilitates understanding of the data by coaches and athletes, hence the integration of the results of development projects into the training and competition routines [18,19,20]. Therefore, starting blocks integrated with force plates and fixed camera systems are commonly used to assess start, turn and swimming performance [21,22,23,24,25]. This technology can be applied in a normal pool environment without interfering with the swimmer’s natural movement patterns. However, the disadvantages are as follows: (1) high purchase costs and (2) immobility of the specific starting blocks and camera systems. Additionally, mounting specific racks and brackets to the pool deck usually requires drilled holes and screws [21,22,23,24], making temporary installations at public pools impractical. Consequently, this technology is typically restricted to an exclusive group of swimmers at high-performance centers, limiting its use in broader applications such as developing young talent at the club level [26].

A major challenge for research and development projects as well as academic works, such as Bachelor theses and testing of new equipment, is the recruitment of homogenous groups of high-level swimmers available at a particular test site [27]. A mobile performance analysis system, which requires minimal equipment and can be mounted in any pool facility, facilitates such research and development projects and the optimal integration of tests into the busy training schedule of elite swimmers. Such systems are commonly used among performance analysts and swimming biomechanists, as proposed by a recent study conducting three-dimensional motion analysis with two sport action cameras, i.e., GoPro [28,29]. Those small cameras are a potential solution to collect high-quality video footage in the swimmers’ natural pool environment. However, the system proposed by Bernardina et al. [28,29] has not yet been validated against the current gold standard for start, turn and swimming analyses nor has its reliability been determined within a group of elite swimmers. Moreover, for the practical use of such a system, percentiles for each parameter provide reference values to identify individual swimmers’ strengths and weaknesses within the start, turn and clean swimming trials. Percentiles rather than mean values provide normative data that cover the entire range of performance levels within a group of athletes [30,31,32].

To permit the collection of quantitative data on the start, turn and clean swimming performances in any swimming pool, the aims of the present study were to (1) validate a mobile in-field performance analysis system (PAS) commonly used in swimming based on minimal equipment (sport action camera) against the current gold standard (Kistler starting block equipped with force plates and a 2D camera system; KiSwim), (2) assess the PAS’s interrater reliability and (3) provide reference values to facilitate the practical use of the PAS in elite junior and adult swimmers.

## 2. Materials and Methods

### 2.1. Participants

Members of the Swiss junior and adult national swimming team volunteered for the present study (n = 47; age: 17 ± 4 [range: 13–29] years; World Aquatics Points: 747 ± 100 [range: 527–994]). All participants were tier 3–5 swimmers, with regular participation in international swimming competitions [33], and included medalists at Olympic Games, World and European Championships. All swimmers or legal guardians in the case of minor-aged participants gave their written informed consent to participate in the study, which was pre-approved by the institutional review board of the Swiss Federal Institute of Sport Magglingen (Reg.-Nr. _198_LSP_Born_05_2023) and in accordance with the code of conduct of the World Medical Association (WMA) for studies involving human subjects (Declaration of Helsinki).

### 2.2. Data Collection

Each participant performed two start and turn trials using their main swimming stroke. For the present validation study, trials were critically assessed by three researchers. If the recording in either the KiSwim or PAS may not allow optimal analysis for all of the targeted parameters, this particular trial was excluded. Hence, 23 trials were not analyzed due to non-optimal visibility of the light flash of the starting signal, blurred vision due to bubbles dragged into the water when submerging the camera or operator error. For each start trial, the participants performed a block start with a subsequent clean swimming phase until 25 m. For each turn trial, the swimmers started 20 m from the pool wall, accelerated to race pace, executed the turn and swam back past the 15 m mark. Time was measured from 5 m before until 10 m after wall contact. A 4- to 5-min rest period between the trials assured sufficient recovery [30]. Before the test session, the swimmers performed a 20-min dry-land warm-up (activation and mobilization exercises) followed by a 15-min in-the-water warm-up (aerobic endurance, technical drills and short sprints).

All trials were video-recorded and analyzed using two methods: (a) the PAS and (b) the KiSwim (Kistler, Winterthur, Switzerland). The KiSwim is a starting block with force sensors (Type 9027C/9028C, Kistler, Winterthur, Switzerland) and a 2D motion analysis system. The KiSwim involves five fixed-view cameras (Prosilica GC660C; Allied Vision Technologies, Stadtroda, Germany) in waterproof casings positioned at the side of the pool perpendicular to the swimming lanes. Video footage was collected at 100 fps. Four underwater cameras were positioned 0.95 m below the water surface at 1.5 m, 5 m, 10 m and 15 m from the pool’s head wall (where the starting block is mounted). At 1.5 m, an additional overwater camera is positioned 0.95 m above the water surface. Cameras and force plates were synchronized to the starting signal (Infinity Start System; Colorado Time Systems, Loveland, CO, USA). For the calibration, a specific calibration rack with seven vertically arranged landmarks from 1.5 m below to 1.5 m above the water surface is moved along the swimming lane and the landmarks are digitized at each 1-m interval. Distance from the head wall is determined with a laser-beam distance measure (Disto D2; Leica Geosystems, Inc., Heerbrugg, Switzerland). Details on the calibration procedure and high reliability of the system have previously been presented [23].

**Figure 1:**
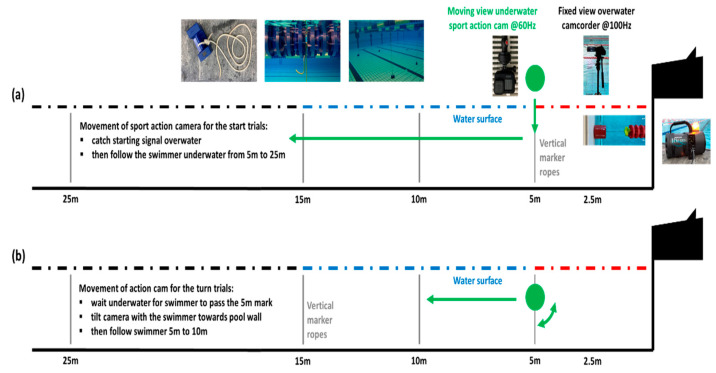
Set-up and camera path of a sport action camera to capture (**a**) start and (**b**) turn trials.

The PAS involves one fixed view camera (FDR-AX700E; Sony, Tokyo, Japan) that is positioned 2.5 m from the pool’s head wall. The camera captures the first 5 m of the swimming lane including the light flash from the starting device, the entire starting block and the aerial trajectory of the flight phase at a frame rate of 100 Hz. A sport action camera (GoPro 8, GoPro, San Mateo, CA, USA) is moved along the side of the pool perpendicular to the swimming lane on a 1.55 m long monostand (Monostand Smooth, Hama, Monheim, Germany) to capture underwater footage. Vertical marker ropes are fixed between weights on the pool floor and the swimming lane ropes at both sides of the test lane at 5 m, 10 m, 15 m and 25 m from the pool’s head wall. The exact positions were determined with a measuring tape and were regularly checked throughout the test session. To prevent movement of the buoys at the lane ropes throughout the test session, all buoys at the test lane are pushed to one side of the pool and fixed with clamps. Swimmers are instructed and closely watched not to pull at the lane ropes, which swimmers tend to do, especially when swimming backstroke at an easy recovery pace. To capture the start trials, the sport action camera is positioned at the 5 m mark. After capturing the light flash of the starting signal, the camera is submerged to about 20 cm below the water surface and moved underwater alongside the swimmer until 25 m. To capture the turn trial, the camera is positioned at 5 m under the water surface, tilted with the movement of the swimmer in the direction of the pool’s head wall and then moved alongside the swimmer until 10 m. Figure 1 illustrates the test set-up and camera paths.

### 2.3. Data Analysis

The data collected with the KiSwim are analyzed with customary software (version 10.0, Kistler Performance Analysis System–Swimming Starts and Turns). The postprocessing of the video footage is semiautomated with the following data points being manually digitized: center of the head at starting position, toe-off, maximal swimming depth, first underwater kick; top of the head at water entry, breakout, −5 m, 5 m, 7.5 m, 10 m, 15 m from the pool wall; center of gravity at toe-off; far side and rear edge of the entry hole. Data collected with the PAS are analyzed with the Kinovea motion analysis software (version 0.9.5, Joan Charmant & Contrib., kinovea.org). Over- and underwater footage are synchronized to the light flash of the starting signal. Flight distances were calibrated to the distance between the pool wall and the 5 m mark, which was carefully checked with a measuring tape before and during the tests. Breakout distances were determined based on the number of floats on the lane ropes with an accuracy of a 10th of a meter as commonly used in race analyses [2,34]. A detailed description of all parameters is provided in Table 1. Timestamps that are manually digitized in the motion analysis software are imported to a specific Excel spreadsheet (Excel 365, Microsoft Corporation, Redmond, DC, USA) to calculate the parameters, i.e., split times, velocities, stroke and kicking rates (refer to Supplementary Material File S1).

To determine validity, each start and turn trials were analyzed with the KiSwim and PAS by a single experienced performance analyst. To investigate the robustness and feasibility of the PAS including procedures, manuals and descriptions, a coach with experience and background in competitive swimming was trained to conduct the analysis for the interrater reliability. A manual with a detailed description of the test procedure and data analysis is provided in the Supplementary Material File S2. The 10th, 25th, 50th, 75th and 90th percentiles were calculated for all parameters with acceptable validity or reliability to provide reference values for start, turn and clean swimming performances. Percentiles specify the position of a data point within a data set as a percentage of the parameter values arranged in a numeric order. Percentiles were calculated for all swimming strokes separately: butterfly, backstroke, breaststroke and freestyle. The five individual medley turns were excluded here.

### 2.4. Statistical Analysis

All data are presented as mean ± standard deviation (SD). The statistical analyses were carried out with JASP statistical software package version 0.16.4 (JASP-Team, University of Amsterdam, Amsterdam, The Netherlands). Normality and linearity were determined with Shapiro–Wilk’s test, Q-Q and scatter plots [35]. If normality was violated or only a monotonic relationship between the two variables was determined, Spearman’s instead of Pearson’s correlation coefficient was used to validate the PAS against the KiSwim. While 25 start, turn and swimming parameters can be measured with the PAS, 16 of those parameters are also measurable with the KiSwim. Hence, validity was determined for those 16 parameters and interrater reliability of all 25 parameters that can be measured with the PAS. Relationships between the two methods were classified as nearly perfect, very large, large, medium, small and trivial with a correlation coefficient of >0.90, 0.90–0.70, 0.70–0.50, 0.50–0.30, 0.30–0.10 and <0.10 [36]. Relative validity was accepted with nearly perfect or very large correlations between the PAS and the KiSwim as well as a %-difference < 1%. Additionally, agreement between the two methods was assessed using Bland–Altman plots showing 95% limits of agreement (1.96 × SD) across the entire range of performance levels of junior and adult national team members [37]. Interrater reliability of the PAS was determined with an intra-class correlation coefficient (ICC). The ICC was calculated according to Shrout and Fleiss [38] with each rater analyzing all participants [ICC(2,B)]. ICC of >0.90, 0.90–0.75, 0.75–0.50 and <0.50 indicated excellent, good, moderate and poor interrater reliability, respectively [39], with relative reliability being accepted with ICC > 0.75 [40,41,42]. A post hoc power analysis was performed with G*Power (version 3.1.9.7) using the bivariate normal model with a two-tailed correlation. An alpha error probability set to 0.05, a sample size of 47, correlation ρ H_1_ at the lowest significant correlation (*r* = 0.49 from the Take-off angle) and the correlation ρ H_0_ at 0, the calculated statistical power was 0.95.

## 3. Results

**Figure 2:**
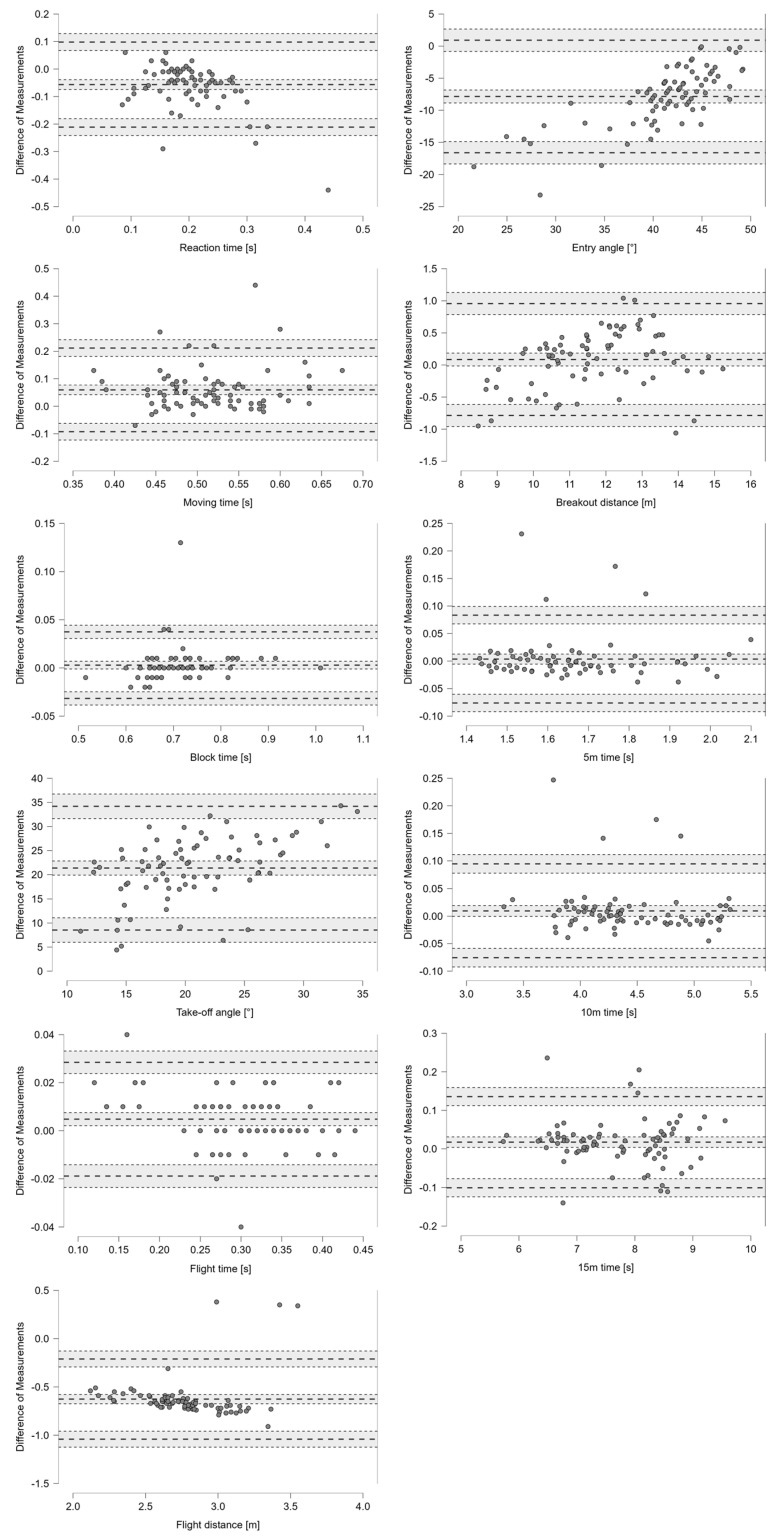
Validity analysis for start performance using Bland–Altman plots with a 95% confidence interval for the difference between the methods (PAS—KiSwim values) and limits of agreement. Values on the x-axis show the means of the two methods.

**Figure 3:**
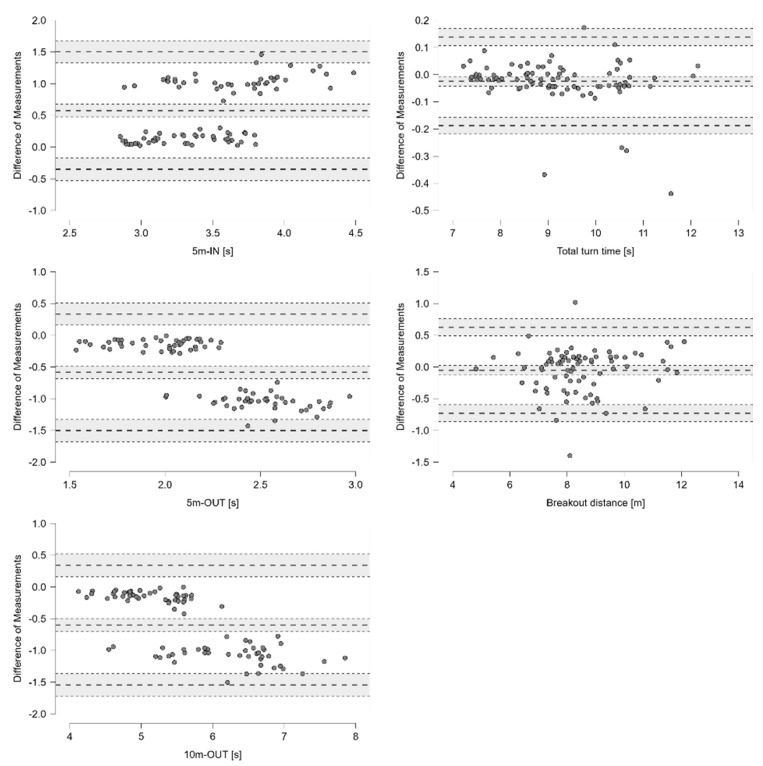
Validity analysis for turn performance using Bland–Altman plots with a 95% confidence interval for the difference between the methods (PAS–KiSwim values) and limits of agreement. Values on the x-axis show the means of the two methods.

Of a total of 25 parameters determined with the PAS, 16 were also measurable with the KiSwim, of which 7 parameters showed satisfactory validity (*r* = 0.95–1.00, *p* < 0.001, %-difference < 1%). As such, nearly perfect correlations (*p* < 0.001) and very small mean differences were evident between the PAS and the current gold standard (KiSwim) for five start parameters, i.e., Block time [s], Breakout distance [m], 5 m, 10 m and 15 m time [s], and two turn parameters, i.e., Total turn time [s] and Breakout distance [m] (Table 2). The remaining parameters had large correlations (*p* < 0.001), with the exception of the Take-off angle [°]. Bland–Altman plots revealed that the PAS underestimates (shows a shorter) Reaction time [s] and overestimates the Moving time [s], while the PAS overestimates (shows a larger) Take-off angle [°] and underestimates the Entry angle [°]. In regard to turn performance, the PAS overestimates (shows a longer) 5 m-IN [s] time but underestimates 5 m-OUT [s] and 10 m-OUT [s] (Figure 2 and Figure 3).

Interrater reliability was determined for all 25 parameters of the PAS, of which 21 parameters showed acceptable relative interrater reliability (ICC > 0.75). While 18 parameters even showed excellent reliability (ICC > 0.90), Kicking rate [bpm] and Distance per kick [m] only showed good interrater reliability (ICC = 0.78–0.85). Reaction time [s], Moving time [s], Flight distance [m] and Pivot time [s] showed unsatisfactory interrater reliability (ICC < 0.75, Table 3). Reference values (10th to 90th percentiles) were provided for all parameters with acceptable validity or reliability (Table 4).

## 4. Discussion

To permit the collection of quantitative data on start, turn and clean swimming performances in any swimming pool, the aims of the present study were to (1) validate a mobile in-field PAS commonly used in swimming based on minimal equipment (sport action camera) against the current gold standard the Kistler starting block equipped with force plates and a 2D camera system (KiSwim), (2) assess the PAS’s interrater reliability and (3) provide reference values to facilitate the practical use of the PAS in elite junior and adult swimmers.

Seven parameters of the PAS showed satisfactory validity from a total of 16 parameters that could be validated against the KiSwim. Additionally, interrater reliability was accepted for 21 parameters of the PAS from a total of 25 start, turn and swimming parameters. The percentiles provide reference values for all parameters for junior and adult national team swimmers across the various swimming strokes. The analysis template and manual provided in the Supplementary Material aid the practical application of the PAS in research and development projects as well as academic works.

Validity and reliability were not accepted for reaction and moving times. With the PAS, the end of reaction time and beginning of moving time were determined by the first visible movement of the swimmer after the starting signal. The visual inspection and lower sampling rate (100 fps video footage) resulted in a larger variety of the PAS data (refer to Bland–Altman plot in Figure 2) and explain the differences to the reaction and moving time determined with the KiSwim based on kinetic data sampled with 500 Hz. However, block time determined by the PAS showed nearly perfect validity and reliability based on objective and reproducible criteria, i.e., a light flash of the starting signal and the last frame at which the toe was still in contact with the starting block. Based on the present findings and previous studies showing little differences in reaction time between swimmers of various performance levels [43], applicants of the PAS should use block time rather than reaction time or moving time for valid and reliable assessment of on-block start performance.

While total turn times showed nearly perfect validity and excellent reliability, 5 m-IN and 5 m-OUT split times showed excellent reliability, yet unsatisfactory validity. Bland–Altman plots (refer to Figure 3) showed that the PAS overestimated 5 m-IN split times and underestimated 5 m-OUT split times compared to the gold standard (KiSwim). The visual inspection of the PAS based on video data recorded at 60 fps determined wall contact later compared to the kinetic data of the KiSwim, which were sampled at 500 Hz. This explains the drift toward longer wall entry and shorter wall exit parameters. However, 5 m-IN and 5 m-OUT split times determined by the PAS were highly reliable and can therefore be used to describe longitudinal performance development between training phases or for intervention studies. Additionally, data from such performance analyses are often used to explain observations from race analyses and determine an individual swimmer’s strengths and weaknesses. Kinematic data retrieved from race analyses are also based on visual inspection of video footage, i.e., wall contact during turns [8,9], and the 5 m-IN and 5 m-OUT split times are therefore comparable with data determined with the PAS.

Take-off and entry angles showed excellent reliability as well. However, the unsatisfactory validity may result from different methodologies used to determine those angles with the PAS and KiSwim. The KiSwim calculates take-off angles from vertical and horizontal force production as well as parabolic flight trajectories. This method represents the whole-body take-off angle including potential decoupled inclination of the upper body and arm movements below or aside the upper body. On the other hand, visually determined take-off angles by the PAS based on body landmarks at toe-off match the coaches’ observations from the video footage and enhance the practical value of the PAS. Marking specific body landmarks with reflective markers would help digitize the video footage and further improve the accuracy. However, reflective markers are time-consuming to attach and impair fluid dynamics as well as swimming performance [44]. Markerless or even automated tracking of specific body landmarks is a future solution [45,46], which, however, was not in alignment with the aims of the study to investigate the validity and reliability of a commonly used mobile in-field performance analysis system to assess start, turn and swimming performance.

From the 21 parameters for which reliability was accepted, kicking rate and distance per kick were only rated with good reliability. The analysis determines the exact same start and end point of the underwater kick, which is less definite with the single underwater kick that is allowed for breaststroke [47]. The large number of breaststroke trials (29%) may have therefore resulted in a larger variation of those parameters. Underwater phases from the other swimming strokes involve a larger number of kicks, such as 7.0 ± 1.6 and 5.7 ± 2.1 for butterfly starts and turns, respectively [48]. Since the first underwater kick is typically affected by the deviation of the underwater trajectory from water entry to a horizontal direction of travel, particularly for those swimmers who start kicking too early and at 4.1 ± 0.5 m [49], the second kick should be used for a reliable analysis of kicking rate and distance per kick with the PAS.

Percentiles are a commonly used method to provide reference values within peer groups exhibiting a diverse range of performances [31,50,51,52,53]. Unlike mean values ± SD [32], percentiles encompass the entire spectrum of performances within a specific sample of athletes, such as national team swimmers. Thereby, percentiles offer comprehensive comparative data to assess and interpret test results, particularly for longitudinal test series spanning one or multiple seasons. Moreover, percentiles enable a relative evaluation of performance indicators, allowing the comparison of test parameters between different race sections, i.e., start, turn and clean swimming phases. This capability facilitates the identification of individual swimmer’s strengths and weaknesses within the large variety of key performance indicators, hence allowing objective goal setting and identification of future potentials [31].

### Limitations of the Study

For the practical application of the PAS, users need to distinguish between valid and reliable parameters. While missing validity limits the comparison of individual swimmers’ data to other studies or methods, the high reliability of those parameters still allows the assessment of longitudinal performance development. Other than visual methods, i.e., force plates, the combination of the PAS with IMU-based sensor technology may further help determine the components of block time, i.e., reaction and moving time. While the performance analysis data from the present study were determined based on isolated start and turn trials, which provide distinguishing factors for the outcome of modern swim races [8,17], the PAS can be expanded to the entire pool length with split times beyond the 25 m mark. This would allow start and turn assessment on both pool sides when conducting a complete race simulation. Again, wearable sensor technology, which has particularly validated for the assessment of technique aspects and parameters during the clean swimming sections [14,15,16], could be combined with the PAS and would provide additional valuable information. Finally, a limitation of the PAS relates to the rate of measurement error. In total, approximately 11% of data acquisitions were not analyzed due to non-optimal visibility of the light flash of the starting signal or blurred vision due to bubbles dragged into the water when submerging the camera. Considering these rates of measurement error, when conducting performance analysis in closed settings practitioners are encouraged to perform multiple trials to reduce the likelihood of complete data loss. Further, these error rates might be reduced with further experience utilizing the PAS.

## 5. Conclusions

In conclusion, the in-field PAS provides a mobile performance analysis system to investigate start, turn and swimming performance with high validity and reliability. Users of the PAS must distinguish between parameters that can be well compared with test results from other methods (validity) and parameters that show high consistency between multiple tests using the PAS. As such, low validity limits the comparability of angles at take-off and during water entry to other analysis methods. Additionally, visual determination of the wall contact when investigating turns with the PAS results in a drift toward longer 5 m-IN and shorter wall exit times, compared to the force plate-based method of the KiSwim. However, high reliability still allows the use of those parameters for the determination of individual performance development across multiple tests and to compare those data to other video-based applications (race analyses). The percentiles provide reference values for elite junior and adult swimmers and should be used to assess individual test results and establish development guidelines.

## Figures and Tables

**Table 1:** Parameter list with explanations for the data analysis.

Nr.	Parameter	Explanation
	**Start performance**	
1	Reaction time [s]	Light signal until first visible movement [seconds]
2	Moving time [s]	First visible movement until toe-off [seconds]
3	Block time [s]	Light signal until toe-off [seconds]
4	Take-off angle [°]	Angle between trochanter major, edge of the starting block and horizontal line at toe-off [degree]
5	Flight time [s]	Toe-off until the top of head on the water surface [meters]
6	Flight distance [m]	Pool wall to top of head on water surface [meters]
7	Entry angle [°]	Angle between trochanter major, top of head (on water surface) and horizontal line along the water surface [degree]
8	Kicking rate [bpm]	One complete kicking cycle during underwater phase [beats per minute]; 60/time of one kicking cycle
9	Distance per kick [m]	Distance covered with one complete kicking cycle during underwater phase [meters]; time of one kicking cycle × velocity
10	Breakout distance [m]	Pool wall until top of head breaks through water surface [meters]
11	Stroke rate [bpm]	One complete arm cycle during clean swimming phase [beats per minute]; 60/time of one arm cycle
12	Distance per stroke [m]	Distance covered with one complete arm cycle during clean swimming phase [meters]; time of one arm cycle × velocity
13	Swimming velocity [m·s^−1^]	Velocity during clean swimming phase (15 m to 25 m mark) [meters per second]
14	5 m time [m]	Light signal until top of head at 5 m mark [meters]
15	10 m time [m]	Light signal until top of head at 10 m mark [meters]
16	15 m time [m]	Light signal until top of head at 15 m mark [meters]
17	25 m time [m]	Light signal until top of head at 25 m mark [meters]
	**Turn performance**	
18	5 m-IN [s]	Split time: top of head at 5 m before the wall until first contact with wall [seconds]
19	5 m-OUT [s]	Split time: first contact with wall until top of the head at 5 m after the wall [seconds]
20	10 m-OUT [s]	Split time: first contact with wall until top of the head at 10 m after the wall [seconds]
21	Total turn time [s]	Top of head at 5 m before until 10 m after the wall
22	Pivot time [s]	Flip turn: initiation of rotation (pitching of head) until first contact of feet with wall; open turn: first contact of hands/fingers at the wall until first contact of feet with wall
23	Kicking rate [bpm]	Refer to start parameters
24	Distance per kick [m]	Refer to start parameters
25	Breakout distance [m]	Refer to start parameters

**Table 2:** Validity analysis for start and turn performances of the PAS compared to the current gold standard, i.e., starting block with force plates and 2D motion analysis system (KiSwim). Spearman’s instead of Pearson’s correlation coefficient was used for non-normally distributed and monotonic data. Agreement was quantified with the %-difference between both methods and the limits of agreement (LOA) ± 1.96 × standard deviation.

Variables	Correlation Coefficient (*r*)	%-Difference	LOA	
			Upper	Lower
Start performance				
Reaction time [s]	0.57 ***	−80.4	572.5	−733.2
Moving time [s]	0.54 ***	10.1	33.8	−13.5
Block time [s]	0.95 ***	0.34	4.97	−4.29
Take-off angle [°]	0.49 ***	67.7	97.1	38.3
Flight time [s]	0.98 ***	2.00	11.78	−7.77
Flight distance [m]	0.90 ***	−26.2	−10.9	−41.5
Entry angle [°]	0.69 ***	−25.6	25.2	−76.5
Breakout distance [m]	0.96 ***	0.47	8.26	−7.31
5 m time [s]	0.96 ***	0.19	4.82	−4.43
10 m time [s]	1.00 ***	0.23	2.22	−1.77
15 m time [s]	1.00 ***	0.23	1.78	−1.31
Turn performance				
5 m-IN [s]	0.50 ***	14.1	35.6	−7.3
5 m-OUT [s]	0.60 ***	−29.9	17.2	−77.1
10 m-OUT [s]	0.89 ***	−10.79	5.87	−27.45
Total turn time [s]	1.00 ***	−0.25	1.39	−1.89
Breakout distance [m]	0.95 ***	−0.75	7.68	−9.19

**Note.** Level of significance of correlation coefficients: *** *p* < 0.001.

**Table 3:** Interrater reliability analysis for start and turn performances of the PAS with intra-class correlation coefficient (ICC) including upper and lower 95% confidence interval (CI).

Variables	ICC	95% CI	
		Upper	Lower
Start performance			
Reaction time [s]	0.25	0.56	−0.10
Moving time [s]	0.43	0.74	−0.10
Block time [s]	0.99	1.00	0.99
Take-off angle [°]	0.94	0.96	0.91
Flight time [s]	0.99	1.00	0.86
Flight distance [m]	0.57	0.80	0.01
Entry angle [°]	0.92	0.96	0.85
Kicking rate [bpm]	0.90	0.94	0.86
Distance per kick [m]	0.84	0.90	0.76
Breakout distance [m]	0.95	0.98	0.86
Stroke rate [bpm]	0.96	0.97	0.94
Distance per stroke [m]	0.97	0.98	0.96
Swimming velocity [m/s]	1.00	1.00	0.99
5 m time [s]	0.94	0.97	0.89
10 m time [s]	0.99	0.99	0.99
15 m time [s]	1.00	1.00	0.99
25 m time [s]	1.00	1.00	1.00
Turn performance			
5 m-IN [s]	0.99	0.99	0.96
5 m-OUT [s]	0.92	0.96	0.78
10 m-OUT [s]	0.99	1.00	0.98
Total turn time [s]	1.00	1.00	1.00
Pivot time [s]	0.53	0.76	0.05
Kicking rate [bpm]	0.85	0.90	0.77
Distance per kick [m]	0.78	0.85	0.67
Breakout distance [m]	0.98	0.99	0.96

**Table 4:** Reference values from the present study’s **freestyle** start and turn trials (n = 55) determined with the PAS for those variables with acceptable validity or reliability using the 10th to 90th percentiles. Reference values for the other swimming strokes are provided in the Supplementary Material (Files S3–S5).

Variables	Freestyle Percentiles [World Aquatics Points]				
	10th	25th	50th	75th	90th
	[679]	[696]	[747]	[780]	[830]
Start performance					
Block time [s]	0.86	0.82	0.73	0.71	0.67
Take off angle [°]	23.6	27.4	30.1	35.5	40.3
Flight time [s]	0.25	0.26	0.31	0.34	0.35
Entry angle [°]	34.4	35.7	38.7	41.6	44.4
Kicking rate [bpm]	143.9	145.3	156.5	163.5	175.5
Distance per kick [m]	0.62	0.68	0.73	0.79	0.86
Breakout distance [m]	8.55	9.88	10.80	12.17	13.00
Stroke rate [bpm]	47.3	52.1	54.1	58.5	62.5
Distance per stroke [m]	1.75	1.82	1.89	1.96	2.06
Swimming velocity [m·s^−1^]	1.59	1.64	1.69	1.83	1.88
5 m time [s]	1.88	1.68	1.64	1.57	1.49
10 m time [s]	4.86	4.37	4.28	4.07	3.83
15 m time [s]	8.09	7.43	7.12	6.69	6.54
25 m time [s]	14.39	13.54	13.00	12.14	11.84
Turn performance					
5 m-IN [s]	3.67	3.52	3.19	3.00	2.94
5 m-OUT [s]	2.16	2.08	1.97	1.82	1.70
10 m-OUT [s]	5.53	5.39	5.07	4.79	4.58
Total turn time [s]	9.23	8.84	8.26	7.74	7.57
Kicking rate [bpm]	104.8	109.0	120.0	128.0	139.2
Distance per kick [m]	0.67	0.70	0.81	0.88	0.96
Breakout distance [m]	6.62	7.10	7.90	8.60	8.84

## Data Availability

All data are available on request by the corresponding author and can be retrieved from the following data repository: https://doi.org/10.5281/zenodo.13854056.

## Supplementary Materials

The following supporting information can be downloaded at https://www.mdpi.com/article/10.3390/s24227186/s1, File S1 Analysis Template; File S2 Manual; File S3 Butterfly percentiles, File S4 Backstroke percentiles; File S5 Breaststroke percentiles (Files S3–S5 are the corresponding reference values to the Freestyle percentiles in Table 4).
