# Supplementary material for: Determining Validity and Reliability of an In-Field Performance Analysis System for Swimming
Source: Sensors (Basel). 2024 Nov 9;24(22):7186. doi: 10.3390/s24227186 (PMC11598412; doi:10.3390/s24227186)
Supplement: Supplementary file 1 [file sensors-24-07186-s001.zip › File S2_Manual PAS.pdf]

Description:
Mobile in-field performance analysis system (PAS)

Performance analysis with sport action cameras

The in-field PAS provides a mobile performance analysis system to investigate start, turn and swimming performance with high reliability and validity when compared to the current gold standard, i.e. force plate equipped starting blocks with 2D camera systems.

More information on validity and interrater reliability can be found in:

Born, D. P.; Polach, M.; Staunton, C., Determining validity and reliability of an in-field performance analysis system for swimming. *Sensors* **2024**, Under review.

Equipment – check list

- 8 ropes with a length of 2.5m
- 8 weights of 5-10 kg
- 2 cameras: 1 sport action camera; the 2nd camera can be any camcorder that can collect video footage at 100 fps
- SD-card reader (if not integrated in the laptop)
- tripod to mount the camcorder
- monostand (microphone stand) of 1.5 m to attach the sport action camera
- starting device with light flash
- measuring tape of 25 m
- computer with the Kinovea motion analysis software (https://www.kinovea.org/download.html) and Microsoft Excel

Installations

- fix lane ropes to prevent buoys on the lane ropes from moving (Pictures [1-2])

- fix vertical marker ropes to weights and lane ropes at exactly 5 m, 10 m, 15 m and 25 m from the pool's head wall; use measuring tape to find the exact positions (Pictures [3-5]); add rubber mats underneath the weights to prevent potential corrosion of the pool floor

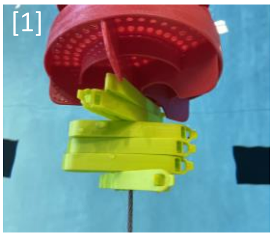
[1]

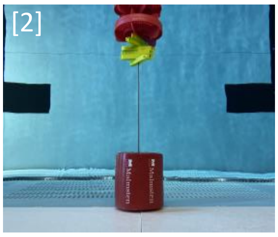
[2]

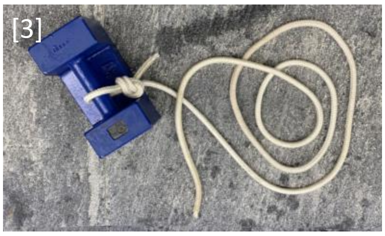
[3]

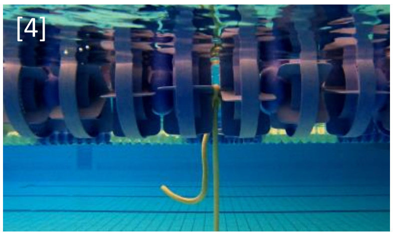
[4]

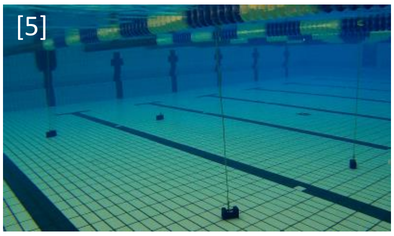
[5]

swiss aquatics swimming
HALMSTAD UNIVERSITY
UMIM PLAVAT.CZ
EHSM Eidgenössische Hochschule für Sport Magglingen

Description:
Mobile in-field performance analysis system (PAS)

- position starting device near the starting block so it is visible from both camera positions (Picture [6])

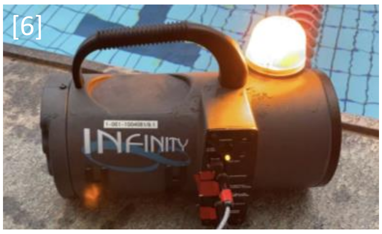
[6]

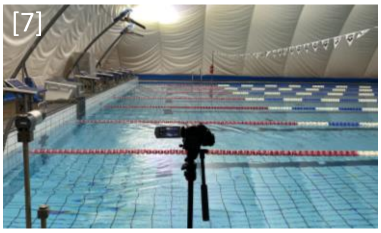
[7]

- set overwater camera to 100 Hz and perpendicular to the lane at 2.5 m from the pool's head wall; assure clear visibility of (Picture [7])
    a) light flash from the starting device
    b) swimmer on starting block and flight trajectory
    c) 0 m and 5 m marks on the lane ropes

[8]

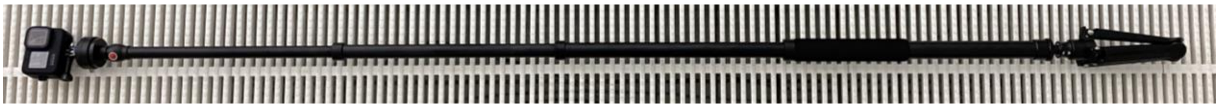

- mount sport action camera on monostand for underwater footage; set framerate to 60Hz (Picture [8])

Procedure (data collection)

Depending on camera zoom settings, use lane 2 or 3 for best results; however, standardize the test lane for all trials. Assure that pool is well lit.

Start
- note time of the day in protocol (facilitates allocation of video footages to each test trial)
- start recording with overwater camera
- start recording with sport action camera
- position sport action camera with clear visibility of starter and light flash at the 5 m mark
- after the start signal, immediately submerge sport action camera at the 5 m mark perpendicular to the swimmer's direction of movement
- follow the swimmer with the sport action camera up to the 25 m mark

Turn
- note time of the day in protocol
- start recording with sport action camera
- position the sport action camera underwater and perpendicular to the swimmer's direction of movement at the 5 m mark
- follow swimmer with the sport action camera towards pool wall by tilting camera
- walk with the swimmer from the 5 m out to the 10 m out markings while keeping the camera perpendicular to the swimmer

Description:
Mobile in-field performance analysis system (PAS)

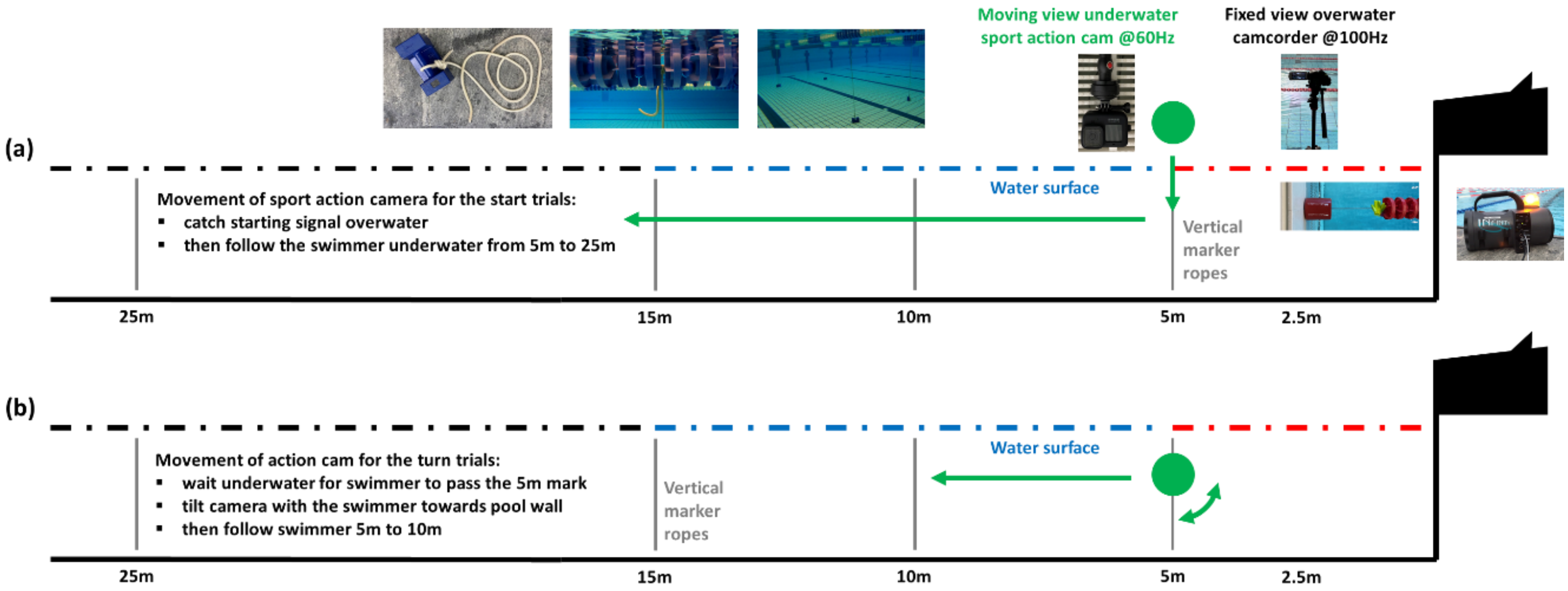

swiss aquatics swimming | HALMSTAD UNIVERSITY | UMIM PLAVAT.CZ | EHSM Eidgenössische Hochschule für Sport Magglingen

Description:
Mobile in-field performance analysis system (PAS)

Tips & tricks

- check clock in cameras and assure correct time settings
- for each trial, note exact time of the day [hh:mm] and potential errors during recording in protocol to facilitate subsequent assignment of videos to the swimmer and trial
- one test person operates overwater camera and starting signal – one test person conducts the filming of the underwater footage
- disable all quick access settings from the sport action camera's video screen (water drop running over the screen may alter your settings)
- swipe up on the sport action camera's screen to activate auto play and to check previously recorded video footage
- if over- and underwater footage will be used with split screen play settings, us 50 Hz for both (possible with GoPro 9+ and newer)
- a video resolution of 1080 Full HD is typically sufficient for visual feedback and digitalization, while minimizing video size for sharing and storing compared to 2.7 or 4K
- the "wide" (16-34mm) lens setting facilitates video capture at high swimming velocities
- shuttle devices such as the Contour Multimedia Controller PRO v2 improves convenience and speed of the analyses: (https://contourdesign.de/produkte/multimedia-controller/contour-multimedia-controller-pro/)

Data analysis (Kinovea)

Check framerate (fps) in the Kinovea task bar and compare it to the camera settings.

Check playback settings in Kinovea at:
_Options
_Time select
_Total milliseconds

Start

- mark key images using the 'Insert' button on the keyboard
- analyze 'Signal' and 'Top of head at 15 m' using the underwater footage for all trials of each individual swimmer
- proceed with fastest trial and mark the key images indicated in the Excel.template for the *overwater* footage
- export timestamps
- repeat the procedure with *underwater* video footage of the same trial

Turn

- determine fastest turn trial by marking 'top of head at 5m-IN and 10m-OUT'
- proceed with fastest trial and mark key images indicated in the Excel.template for the *underwater* footage
- export timestamps

Template (Excel)

- import timestamps from Kinovea to the Excel analysis template

Description:
Mobile in-field performance analysis system (PAS)

Parameters

- **Reaction time [s]**

Light signal until first visible movement [seconds]

- **Moving time [s]**

First visible movement until toe-off [seconds]

- **Block time [s]**

Light signal until toe-off [seconds] (Pictures [9-10])

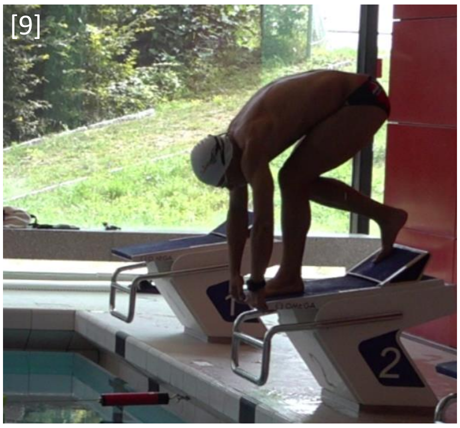

[9]

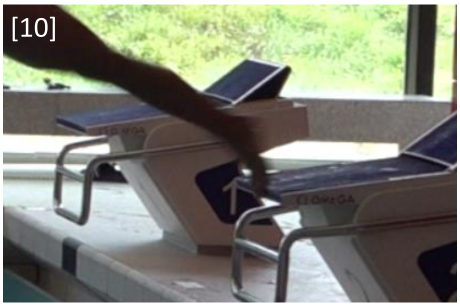

[10]

- **Take-off angle [°]**

Angle between trochanter major, edge of the starting block and horizontal line at toe-off [degree]

- **Flight time [s]**

Toe-off until top of head on water surface [meters]

- **Flight distance [m]**

Pool wall to top of head on water surface [meters] (Picture [11]); calibrate the distance in the key image based on the distance between pool wall and 5m mark

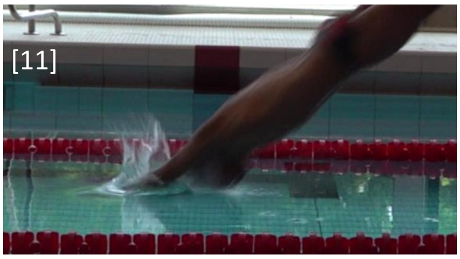

[11]

- **Entry angle [°]**

Angle between trochanter major, top of head (on water surface) and horizontal line along the water surface [degree]

- **Kicking rate [bpm]**

One complete kicking cycle during underwater phase [beats per minute]; 60 / time of one kicking cycle; use 2nd underwater kick in butterfly, backstroke and freestyle for most reliable results

- **Distance per kick [m]**

Distance covered with one complete kicking cycle during underwater phase [meters]; time of one kicking cycle x velocity

- **Breakout distance [m]**

Pool wall until top of head breaks through water surface [meters]; count number of floats on the lane ropes to calculate breakout distance with an accuracy of a 10th of a meter

Description:
Mobile in-field performance analysis system (PAS)

- **Stroke rate [bpm]**

One complete arm cycle during the clean swimming phase [beats per minute]; 60 / time of one arm cycle; consistently use non-breathing arm cycles for best reliability

- **Distance per stroke [m]**

Distance covered with one complete arm cycle during clean swimming phase [meters]; time of one arm cycle x velocity

- **Swimming velocity [$m \cdot s^{-1}$]**

Velocity during clean swimming phase (15m to 25m mark) [meters per second]

- **5m time [m]**

Light signal until top of head at 5m mark [meters] – (top of the head should be perfectly aligned with both vertical lane ropes or should be perfectly center between them) (Pictures [12-13])

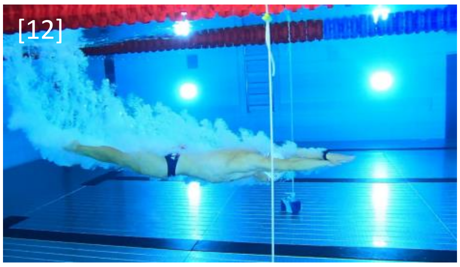

- **10m time [m]**

Light signal until top of head at 10m mark [meters]

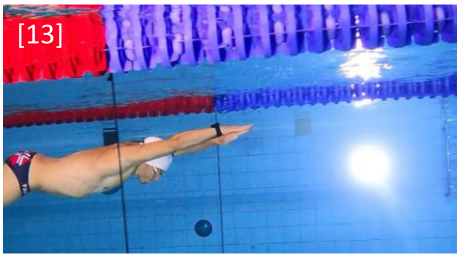

- **15m time [m]**

Light signal until top of head at 15m mark [meters]

- **25m time [m]**

Light signal until top of head at 25m mark [meters]s

- **5m-IN [s]**

Split time: top of head at 5m before the wall until first contact with wall [seconds]

- **5m-OUT [s]**

Split time: first contact with wall until top of the head at 5m after the wall [seconds]

- **10m-OUT [s]**

Split time: first contact with wall until top of the head at 10m after the wall [seconds]

- **Total turn time [s]**

Top of head at 5m before until 10m after the wall

- **Pivot time [s]**

Flip turn: initiation of rotation (pitching of head) until first contact of feet with wall; open turn: first contact of hands/fingers at the wall until first contact of feet with wall
