# Supplementary material for: Determining Validity and Reliability of an In-Field Performance Analysis System for Swimming
Source: Sensors (Basel). 2024 Nov 9;24(22):7186. doi: 10.3390/s24227186 (PMC11598412; doi:10.3390/s24227186)
Supplement: Supplementary file 1 [file sensors-24-07186-s001.zip › File S3_Reference values_Butterfly.pdf]

Supplementary material File S3. Reference values from the present study's **butterfly** start and turn trials (n = 35) determined with the nPASS for those variables with acceptable validity or reliability using the 10th to 90th percentiles.

**Variables**	**Butterfly percentiles** [World Aquatics Points]				
	**10th** [597]	**25th** [658]	**50th** [679]	**75th** [739]	**90th** [868]
**Start performance**					
Block time [s]	0.79	0.76	0.72	0.67	0.66
Take off angle [°]	27.05	30.85	33.65	37.52	41.95
Flight time [s]	0.28	0.29	0.32	0.35	0.40
Flight distance [m]	2.24	2.35	2.44	2.53	2.76
Entry angle [°]	34.70	36.53	37.85	40.65	44.15
Kicking rate [bpm]	124.7	142.5	156.3	165.5	179.6
Distance per kick [m]	0.51	0.68	0.74	0.89	1.04
Breakout distance [m]	9.40	10.38	12.35	13.25	14.75
Stroke rate [bpm]	50.0	56.0	59.5	61.5	64.8
Distance per stroke [m]	1.40	1.62	1.69	1.80	1.84
Swimming velocity [$m \cdot s^{-1}$]	1.41	1.44	1.66	1.67	1.79
5m time [s]	1.83	1.82	1.59	1.54	1.48
10m time [s]	5.19	4.93	4.18	3.95	3.65
15m time [s]	8.58	8.33	7.14	6.82	6.25
25m time [s]	15.70	15.33	13.14	12.76	11.89
**Turn performance**					
5m-IN [s]	4.40	4.10	3.77	3.70	3.62
5m-OUT [s]	2.31	2.06	1.83	1.71	1.59
10m-OUT [s]	6.16	5.55	4.91	4.70	4.55
Total turn time [s]	10.48	9.59	8.82	8.47	8.17
Kicking rate [bpm]	94.4	106.0	124.0	130.5	134.0
Distance per kick [m]	0.67	0.74	0.80	0.90	0.98
Breakout distance [m]	7.14	8.00	8.50	11.40	11.72
