# Supplementary material for: Determining Validity and Reliability of an In-Field Performance Analysis System for Swimming
Source: Sensors (Basel). 2024 Nov 9;24(22):7186. doi: 10.3390/s24227186 (PMC11598412; doi:10.3390/s24227186)
Supplement: Supplementary file 1 [file sensors-24-07186-s001.zip › File S4_Reference values_Backstroke.pdf]

Supplementary material File S4. Reference values from the present study's **backstroke** start and turn trials (n = 22) determined with the nPASS for those variables with acceptable validity or reliability using the 10th to 90th percentiles.

**Variables**	**Backstroke percentiles** [World Aquatics Points]				
	**10th** [719]	**25th** [755]	**50th** [794]	**75th** [874]	**90th** [905]
**Start performance**					
Block time [s]	0.65	0.64	0.63	0.61	0.60
Take off angle [°]	16.40	17.85	20.70	25.40	26.40
Flight time [s]	0.14	0.15	0.18	0.18	0.34
Flight distance [m]	1.88	1.92	1.96	2.28	2.78
Entry angle [°]	16.80	18.70	22.60	27.05	29.10
Kicking rate [bpm]	149.6	150.0	156.7	171.4	179.6
Distance per kick [m]	0.53	0.60	0.61	0.70	0.74
Breakout distance [m]	11.30	11.85	12.90	13.25	13.60
Stroke rate [bpm]	49.3	50.3	52.1	53.3	53.7
Distance per stroke [m]	1.70	1.78	1.84	1.89	1.93
Swimming velocity [$m \cdot s^{-1}$]	1.49	1.49	1.51	1.68	1.72
5m time [s]	2.05	2.00	1.92	1.71	1.55
10m time [s]	5.22	5.21	5.12	4.48	3.94
15m time [s]	8.51	8.45	8.41	7.27	6.57
25m time [s]	15.18	15.16	15.03	13.16	12.41
**Turn performance**					
5m-IN [s]	3.82	3.69	3.47	3.21	3.15
5m-OUT [s]	2.05	2.01	1.92	1.53	1.50
10m-OUT [s]	5.44	5.43	4.97	4.27	4.15
Total turn time [s]	9.26	9.18	8.44	7.43	7.39
Kicking rate [bpm]	120.0	124.0	128.0	128.0	133.0
Distance per kick [m]	0.66	0.67	0.77	0.88	0.90
Breakout distance [m]	7.40	8.20	11.80	12.60	12.90
