# Supplementary material for: Determining Validity and Reliability of an In-Field Performance Analysis System for Swimming
Source: Sensors (Basel). 2024 Nov 9;24(22):7186. doi: 10.3390/s24227186 (PMC11598412; doi:10.3390/s24227186)
Supplement: Supplementary file 1 [file sensors-24-07186-s001.zip › File S5_Reference values_Breaststroke.pdf]

Supplementary material File S5. Reference values from the present study's **breaststroke** start and turn trials (n = 48) determined with the nPASS for those variables with acceptable validity or reliability using the 10th to 90th percentiles.

Variables	Breaststroke percentiles [World Aquatics Points]				
	**10th** [661]	**25th** [687]	**50th** [730]	**75th** [776]	**90th** [890]
**Start performance**					
Block time [s]	0.77	0.74	0.72	0.67	0.67
Take off angle [°]	26.10	28.35	32.25	36.27	41.35
Flight time [s]	0.27	0.28	0.30	0.37	0.40
Flight distance [m]	2.24	2.29	2.40	2.66	2.73
Entry angle [°]	37.25	39.30	41.05	43.38	46.05
Kicking rate [bpm]	53.9	58.8	66.6	81.3	89.8
Distance per kick [m]	1.05	1.23	1.53	1.81	2.05
Breakout distance [m]	10.50	10.90	11.70	12.78	13.75
Stroke rate [bpm]	48.3	49.6	55.8	59.0	63.7
Distance per stroke [m]	1.23	1.33	1.50	1.58	1.67
Swimming velocity [$m \cdot s^{-1}$]	1.24	1.28	1.35	1.40	1.45
5m time [s]	1.74	1.69	1.64	1.53	1.47
10m time [s]	5.15	4.83	4.43	4.26	4.00
15m time [s]	9.13	8.81	8.41	8.14	7.63
25m time [s]	17.21	16.44	15.82	15.17	14.62
**Turn performance**					
5m-IN [s]	4.86	4.66	4.43	4.27	4.11
5m-OUT [s]	2.15	2.11	2.02	1.97	1.83
10m-OUT [s]	6.55	6.23	6.09	5.84	5.44
Total turn time [s]	11.35	10.74	10.48	10.09	9.63
Kicking rate [bpm]	55.5	63.0	71.5	79.3	89.4
Distance per kick [m]	0.88	0.96	1.06	1.17	1.32
Breakout distance [m]	7.50	7.80	8.25	9.10	9.60
